# Regulatory Dendritic Cells for Immunotherapy in Immunologic Diseases

**DOI:** 10.3389/fimmu.2014.00007

**Published:** 2014-01-31

**Authors:** John R. Gordon, Yanna Ma, Laura Churchman, Sara A. Gordon, Wojciech Dawicki

**Affiliations:** ^1^Department of Medicine, University of Saskatchewan, Saskatoon, SK, Canada

**Keywords:** dendritic cell, tolerance, regulatory T cell, immunoregulation, IL-10, retinoic acid, TGFβ, vitamin D

## Abstract

We recognize well the abilities of dendritic cells to activate effector T cell (Teff cell) responses to an array of antigens and think of these cells in this context as pre-eminent antigen-presenting cells, but dendritic cells are also critical to the induction of immunologic tolerance. Herein, we review our knowledge on the different kinds of tolerogenic or regulatory dendritic cells that are present or can be induced in experimental settings and humans, how they operate, and the diseases in which they are effective, from allergic to autoimmune diseases and transplant tolerance. The primary conclusions that arise from these cumulative studies clearly indicate that the agent(s) used to induce the tolerogenic phenotype and the status of the dendritic cell at the time of induction influence not only the phenotype of the dendritic cell, but also that of the regulatory T cell responses that they in turn mobilize. For example, while many, if not most, types of induced regulatory dendritic cells lead CD4^+^ naïve or Teff cells to adopt a CD25^+^Foxp3^+^ Treg phenotype, exposure of Langerhans cells or dermal dendritic cells to vitamin D leads in one case to the downstream induction of CD25^+^Foxp3^+^ regulatory T cell responses, while in the other to Foxp3^−^ type 1 regulatory T cells (Tr1) responses. Similarly, exposure of human immature versus semi-mature dendritic cells to IL-10 leads to distinct regulatory T cell outcomes. Thus, it should be possible to shape our dendritic cell immunotherapy approaches for selective induction of different types of T cell tolerance or to simultaneously induce multiple types of regulatory T cell responses. This may prove to be an important option as we target diseases in different anatomic compartments or with divergent pathologies in the clinic. Finally, we provide an overview of the use and potential use of these cells clinically, highlighting their potential as tools in an array of settings.

During the 1960s, it was thought that macrophages, with their capacity to phagocytose antigens, were required to initiate immunity to foreign substances (1). It was known that lymphocytes were mediators of immunity, but we knew little about how antigens from an invading pathogen would reach the lymph node-sequestered naïve lymphocytes (2). There was a gap in the understanding of the initiation of adaptive immunity, a gap that Ralph Steinman and Zanvil Cohn set out to fill. When Steinman began to study the spleen and lymph nodes, he observed new cells that were distinct from macrophages in appearance and function. These dendritic cells, so named because of their dendrite-like projections, had few lysosomes and only moderate phagocytic activity (3, 4), but they expressed high levels of major histocompatibility complex (MHC) molecules required for presentation of extra-cellular antigens (5). He also observed that dendritic cells were highly potent immune stimulators (6), and now we often speak of dendritic cells as the most proficient of professional antigen-presenting cells (APCs). By 1991, we had accumulated substantial knowledge on the role of the dendritic cell in the induction of immunity, but we were just beginning to recognize that extrathymic dendritic cells could also play central roles in the induction of tolerance (7), and it was not long before we began to understand more about tolerogenic dendritic cells and their potential applications (8–10). We now appreciate that there are numerous discreet populations of naturally occurring regulatory dendritic cells, but focusing on understanding the immunobiology of these cells within their individual niches has given us substantial insights on how we can generate and employ regulatory dendritic cells for immunotherapeutic applications. While dendritic cells can activate either CD4^+^ or CD8^+^, or even CD4^−^CD8^−^ T, B, and NK cells to become regulatory cells, this review will be confined to a discussion on tolerogenic DC in the context of CD4^+^ T cells and their responses. We will first describe the populations of dendritic cells found *in vivo* and then look at the major populations of regulatory dendritic cells that have been induced *ex vivo*, as well as the effector molecules employed by these cells.

## Overview of Dendritic Cell Biology

In general, dendritic cells express MHCII but lack T cell (CD3), B cell (CD19), and NK cell (CD56) lineage markers (11); some subsets of dendritic cells express the monocyte/macrophage (CD14) or NK cell/neutrophil and monocyte/macrophage (CD16) lineage markers, and others the CD4 and/or CD8 T cell subset markers. Dendritic cells are formed from bone marrow progenitors that in general give rise to circulating dendritic cell precursors (12, 13) that seed the peripheral tissues as immature cells (14). As quiescent or immature cells, they express receptors for, and have an innate capacity to respond to an array of inflammatory signals, including ligands for toll-like receptor (TLR), NOD-like receptors, and scavenger receptors, as well as inflammatory mediators, cytokines, and chemokines. The various sub-populations of dendritic cells can respond in a qualitatively and quantitatively distinct fashion to such environmental triggers and differentiate extensively to become immunocompetent accessory cells, such that they provide a crucial link between the innate and adaptive immune responses (15). They upregulate cell-surface expression of their antigen-presentation machinery, including processed antigen peptide-loaded MHCII (16) and co-stimulatory molecules as well as receptors for lymph node-homing chemokines (e.g., CCR7), and they downregulate their phagocytic activities and receptors for local inflammatory signals (e.g., CCR5, CCR6) (14, 17). As dendritic cells mature, they lose their ability to process new peptides (18, 19) and migrate to their tissue-draining lymphoid organ, where they present their processed antigens to T cells in the context of cell-surface MHC (APC signal 1) together with supporting co-stimulatory molecules (e.g., CD40, CD86; APC signal 2) and T cell-polarizing cytokine signals such as IL-12 (20) (APC signal 3), inducing the T cells to differentiate into antigen-specific effector T cells (Teffs; e.g., Th1, Th2, or Th17 cells) (13). But dendritic cells can also provide a fourth APC signal of sorts to T cells, by which they direct the trafficking of the educated T cell. In the gut, retinoic acid and transforming growth factor (TGF)-β produced by dendritic cells together induce T cells to express the α4β7 and CCR9 gut-homing receptors (21), while in the skin-draining lymph nodes vitamin D metabolites released by the dendritic cell induce T cells to express CCR10, such that they become responsive to the skin-homing chemokine CCL27 (22).

**Figure 1:**
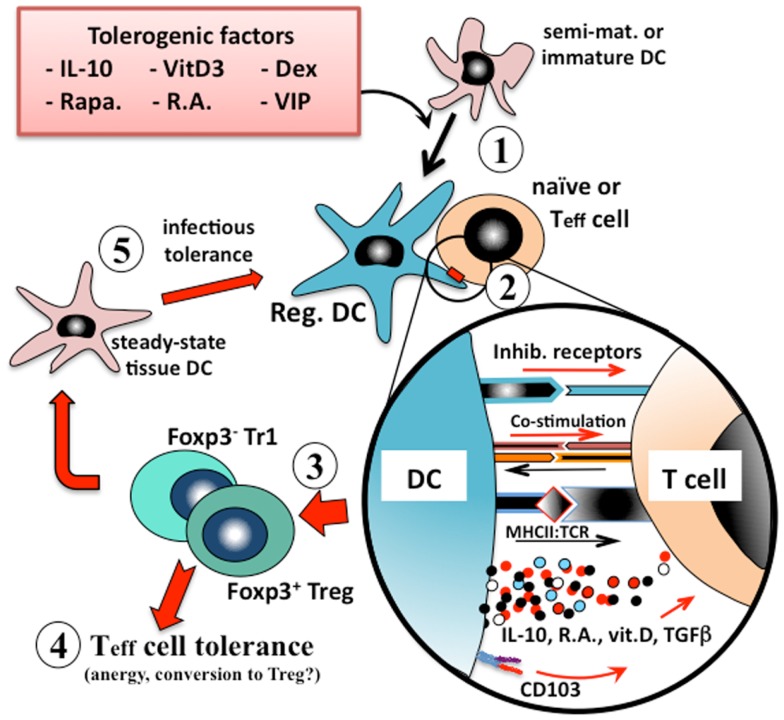
**Induction of immunologic tolerance by regulatory dendritic cells**. Immature or semi-mature dendritic cells that are incubated with, or differentiated in the presence of, tolerogenic factors (e.g., IL-10, vitamin D3, corticosteroids, or retinoic acid) (1) adopt a regulatory phenotype. When these converted regulatory dendritic cells are pulsed with antigen and exposed to cognate naïve or effector T (Teff) cells (2), they present their processed antigen peptides in the context of MHCII, and also lower levels of co-stimulation (e.g., CD40, CD86) to the T cells, but at the same time many types of tolerogenic cells also provide inhibitory receptor (e.g., ILT2, ILT4) signaling to the T cell. Counter-signaling from the engaged T cell activates dendritic cell production of polarizing mediators (e.g., IL-10, TGFβ), which together instruct the T cell to adopt a regulatory phenotype. The nature of the instructional signals from the dendritic cell to the T cell determine whether it adopts an IL-10-secreting CD25^+^Foxp3^+^ Treg phenotype or an IL-10/TGFβ-secreting Foxp3^−^ Tr1 phenotype (3). These regulatory T cells are able to suppress the responses of cognate or by-stander naïve or effector T cells in their microenvironment (4) and also to convert endogenous tissue dendritic cells to adopt a regulatory phenotype through induction of infectious tolerance (5), and thereby reinforce the tolerance phenotype.

Tissue-resident dendritic cells that acquire innocuous environmental or self antigens in the absence of local inflammatory responses similarly migrate to the draining lymph nodes but, as more quiescent cells, overall they express lower levels of MHCII, co-stimulatory molecules, and IL-12, and secrete instructional regulatory mediators such as IL-10 or retinoic acid (23, 24). In this way dendritic cells that are presenting innocuous environmental antigens activate one of several types of regulatory T cell (e.g., Treg, Tr1, or Th3) responses that are associated with immune tolerance (Figure 1).

## Naturally Occurring Populations of Dendritic Cells

A large number of reports have described an array of dendritic cell types and subtypes in different organ systems and animals, and it is almost undoubtedly true that more will be described as we explore further. Many of these sub-populations are or can be tolerogenic as they are found in their steady state (e.g., pulmonary plasmacytoid or myeloid dendritic cells), but for most if not all of these there are inflammatory signals that can override this tolerogenic phenotype, converting these cells to an immunostimulatory phenotype. In some tissues (e.g., gut, liver) dominantly tolerogenic signals are constitutively expressed at high levels, while in other sites that are not routinely exposed to the external environment these signals may be much more subtle.

### Dendritic cells in the blood

Several distinct types of dendritic cells can be identified in human peripheral blood. There are two sub-populations of MHCII^+^CD11c^+^CD123^lo^ myeloid dendritic cells, including CD1c/blood dendritic cell antigen (BDCA)-1^+^ cells and CD141/BDCA-3^+^ cells, as well as MHCII^+^CD1c^−^CD123^hi^ plasmacytoid dendritic cells that also express BDCA-2/CD303, BDCA-4/CD304, IL-3RA, and ILT7 (11, 25). The CD141^+^MHCII^+^CD11c^+^ myeloid dendritic cell is the human counterpart of the murine CD8α^+^ dendritic cell (25). In the mouse, the identities of circulating tissue dendritic cell precursor(s) have not been all that well documented (26). We know that murine splenic and lymph node dendritic cells are continuously replaced from a pool of blood-borne precursors (27), that splenic CD8α^+^ dendritic cells most likely gain access to this organ via the vasculature (28), and that MHCII^lo^CD11c^lo^ pDC do accumulate in the blood of mice (13, 28). While immunostimulatory (29) and tolerogenic (30) dendritic cells can be readily differentiated *ex vivo* from peripheral blood monocytes in humans, it was only recently that LPS stimulation of murine monocytes was reported to induce dendritic cell differentiation (31). These murine monocyte-derived dendritic cells express CCR7 and dendritic cell-specific intracellular adhesion molecule 3-grabbing non-integrin (DC-SIGN) and localize to T cell areas of lymph nodes, where they are highly effective in presenting and cross-presenting antigens (31).

In humans, the BDCA-1^+^ and -3^+^ myeloid dendritic cell populations can be mobilized from the bone marrow with Flt3 ligand alone while optimal plasmacytoid dendritic cells mobilization reportedly calls for use of Flt3 ligand and G-CSF (25). The circulating BDCA-1^+^/CD1c^+^ myeloid dendritic cell can secrete abundant IL-12 and prime cytotoxic T cell responses (32), while BDCA-3^+^ myeloid dendritic cells and BDCA-2^+^ plasmacytoid dendritic cells instead secrete IFNγ and IFNα, respectively, on activation (32). A minor population of tolerogenic IL-10-expressing CD1c^−^CD303^−^CD14^+^ dendritic cells has recently been described in human peripheral blood, although much of the data regarding their tolerogenic activities has come from studies with an *in vitro* analog of the circulating cell (33).

### Intestinal dendritic cells

The intestinal immune system routinely faces the challenge of discriminating pathogens from harmless commensal organisms and other (e.g., food) antigens, as a prelude to triggering effector and regulatory T cell responses, respectively (34). The gut-associated dendritic cells include those in the mesenteric lymph nodes (MLNs), intestinal lamina propria, and the isolated lymphoid follicles (35, 36). The lamina propria contains two populations of CD11c^+^ mononuclear cells, including CD11c^hi^CD103^+^CD11b^+^CX_3_CR1^-^ cells and CD11c^int^CD103^-^CD11b^+^CX_3_CR1^+^ cells; the CD103^+^ cells are *bona fide* dendritic cells while the latter CD103^−^ cells are now thought to be resident tissue macrophages (37). Under steady-state conditions, the CD103^+^ dendritic cells express retinaldehyde dehydrogenase 2 (RALDH2) (23, 38), TGF-β (39), and indoleamine-2,3-dioxygenase (IDO) (40), such that targeting of antigens to these cells leads to tolerance outcomes, while gut inflammation dampens TGFβ and RALDH2 expression in these cells, such that they instead induce vigorous T and B cell responses (41, 42). CD103, the α chain of the E-cadherin ligand αEβ7 integrin (43), is expressed on almost all lamina propria dendritic cells and a subset of MLN dendritic cells (44). It has been reported that gut luminal bacteria recruit lamina propria CD103^+^ dendritic cells into the gut epithelium, from which they extend filipodia into the lumen to sample gut antigens (37). RALDH2 is an enzyme that catalyzes the synthesis of retinoic acid, a vitamin A derivative, which plays a major role in immunologic tolerance within the gastrointestinal tract (45). Expression of CD103 and retinoic acid together induce gut T cells to express the gut-homing receptors CCR9 and α4β7 (44, 46). CCR9 and its CCL25 ligand regulate recruitment of lymphocytes to the vasculature of the small intestine (47), while α4β7 integrin expression confines extravasation of these T cells to the intestinal post-capillary venules (48). Retinoic acid and TGFβ together promote the differentiation of Foxp3^+^ Treg from naive T cells (39), while retinoic acid further reinforces tolerance by dampening Th17 cell differentiation (49). Retinoic acid also fosters B cell isotype switching to IgA antibodies as well as their expression of CCR9 and α4β7 (50–52), and thereby contributes further to local tolerance responses.

### Pulmonary dendritic cells

Pulmonary dendritic cells can be differentially positioned in either the conducting airway or the interstitium of the lung (15, 53). In mice, CD11c^hi^ myeloid cells are found in both compartments, while CD11c^−^ cells are reportedly confined to the airway mucosa (53). The airway dendritic cells form a prototypical network of interdigitating cells positioned beneath the epithelium (54–56), with many of these cells extending dendritic processes into the airway lumen to sample airway antigens (57), just as occurs in the gut (37). In mice these airway cells express CD11c^+^, MHCII^+^, and CD11b^+^, but not CD8α^−^ (15); they also express CD103 and tight junction proteins (claudin-1 and -7, and zonula occludens protein 2), which would play important roles vis-à-vis their positioning within the epithelium (43). After airway antigen sampling and processing, these cells can activate cognate T cells in their immediate environment (57, 58), but also migrate to the lung-draining lymph nodes where they present to T cells in that compartment (58). In rats the airway-associated dendritic cells are somewhat more heterogeneous (53). Bronchoalveolar lavage (59) and tissue digest (60) studies of the human lung have revealed three populations of dendritic cells, including CD11c^+^CD1c^+^ and CD11c^+^BDCA-3^+^ myeloid cells, and CD11c^−^BDCA-2^+^ plasmacytoid dendritic cells, and these are considered analogous to the CD11b^+^CD103^−^ and CD11b^−^CD103^+^langerin^+^ conventional and plasmacytoid dendritic cell subsets, respectively, in mice (61). Further analysis in chronically inflamed (e.g., COPD) lung tissues have revealed langerin-positive and DC-SIGN-expressing dendritic cell sub-populations (31, 62) that were proposed to represent the human equivalent of the murine CD11b^−^CD103^+^langerin^+^ and monocyte-derived inflammatory dendritic cells, respectively (31, 62). The CD103^+^ dendritic cells that comprise the bulk of the dendritic cells found in the lung-draining lymph node migrate there from the lung mucosa under the influence of lymph node-homing chemokines that signal via the CCR7 (43). In humans, the lung plasmacytoid dendritic cells express CD123 and BDCA-2, while the mouse plasmacytoid dendritic cell is B220^hi^Ly6C^hi^Gr1^lo^CD11b^−^CD11c^lo^ (63). Plasmacytoid dendritic cells, which contribute importantly to tolerance responses to innocuous airway antigens (64), also express CD45RA, Ly49Q, BST2/tetherin [or murine plasmacytoid dendritic cell antigen (mPDCA)], sialic acid-binding immunoglobulin-type lectin (siglec)-H, inducible costimulator ligand (ICOS-L), programed death 1 ligand (PD-L)-1, and IDO (65), but produce copious amounts of IFNα in response to viral challenge (66). Under tolerogenic conditions, the CD103^−^ and CD103^+^ dendritic cells reportedly are specialized in presenting antigen to CD4^+^ versus CD8^+^ T cells, respectively (67, 68). However, under viral challenge the CD103^+^ dendritic cells efficiently migrate to the draining lymph nodes where they cross-present viral antigens to CD8^+^ T cells, while the CD103^−^ cells tend to remain within the lung parenchyma, where they present to CD4^+^ T cells in a pro-inflammatory manner (67, 68). This separation of function is also observed in asthmatic animals, wherein the CD103^−^ dendritic cells present allergen to parenchymal CD4^+^ T cells, while the CD103^+^ subset presents allergen in the draining lymph node (69).

It is clear that the pulmonary dendritic cell contributes not only to the induction of asthma, but also to allergen-tolerance. Wholesale depletion of CD11c^+^ cells abolishes disease onset following allergen exposure in experimental animals (70), but plasmacytoid dendritic cell depletion in animals challenged with otherwise innocuous aeroallergens leads to development of allergen-specific asthmatic responses (64). Steady-state plasmacytoid dendritic cells express an immature/semi-mature phenotype, with low levels of MHCII and co-stimulatory molecules and intermediate levels of PDL-1 (15, 71), which would contribute to their tolerogenic phenotype, but IDO expression by these cells also strongly inhibits T cell proliferative responses (72). Nevertheless, CD103^+^ dendritic cells from the lungs of allergen-tolerant mice would also affect tolerance, inasmuch as they express RALDH and secrete retinoic acid, which contributes together with TGFβ to local induction of Foxp3^+^ regulatory T cells (73). Finally, it is important to note the contributions of other populations within the lung to tolerance. Tissue-resident (74) and alveolar (75) macrophages both express TGFβ and RALDH under steady-state conditions, such that they can also induce CD4^+^ T cells to which they present innocuous antigens to convert into Foxp3^+^ Treg. Alveolar macrophages can also suppress the immunostimulatory properties of steady-state lung-resident dendritic cells (76) and thereby further contribute to steady-state tolerance in the lung.

### Cutaneous dendritic cells

As with the intestinal tract and lungs, the skin is constantly exposed both to pathogens, which require induction of protective Teff responses, and to innocuous environmental agents for which tolerance is the desired outcome. There are at least three subsets of skin-derived dendritic cells, including the self-renewing epidermal langerin^+^CD103^−^ Langerhans cell (77), and the langerin^+^CD103^+^ (78, 79) and langerin^−^CD103^−^ (80) dermal subsets; others have reported that the dermis contains five distinct subsets of dendritic cells (81). The epidermal Langerhans cell is probably the best known dendritic cell – as in other interfaces with our environment, these superficial cells form a contiguous network of interdigitating cells that are well positioned to detect and respond to cutaneous insults (82). In general, skin dendritic cells that acquire local antigens for lymph node presentation downregulate their E-cadherin epithelial receptors and upregulate CCR7, thereby acquiring responsiveness to chemokines expressed in the T cell zones of the draining lymph nodes (e.g., CCL19, CCL21) (14). In the lymph node, the dendritic cell presents its processed antigen peptides to the T cell, along with its co-stimulatory and polarizing signals. In addition, vitamin D3 metabolites expressed by the antigen-presenting dendritic cell induces T cell upregulation of CCR10, the receptor for the skin-homing chemokine CCL27 (22).

Langerin^+^ migratory skin dendritic cells (i.e., CD103^+^ dermal dendritic cells and Langerhans cells) can promote T cell tolerance responses to self antigens (83). The Langerhans cell appears to be unique in some respects, however, such that exposure to potent inflammatory adjuvants by itself does not override their innate tolerogenicity (84), perhaps in part because they do not express a number of important microbial pattern recognition receptors (e.g., TLR2, TLR4, or TLR5) (85). They are also unique in that, even while in a tolerogenic mode, they strongly express APC co-stimulatory markers and express IL-12. Nevertheless they fail to effectively activate NF-κB (i.e., translocate RelB into the nucleus) following adjuvant exposure (84), which is critical to induction of the immunostimulatory phenotype in dendritic cells (86). While dermal dendritic cells can effectively induce anti-bacterial immune responses, and would need to do so in situations where microbial organisms successfully penetrate the epithelial barrier, the default function of the Langerhans cell instead leads to regulatory T cell responses, perhaps as a means of preventing the integrity of the epidermal barrier from being compromised (85). Indeed, Langerhans cell depletion (e.g., by UV-B light exposure) has long been recognized to augment pathology in multiple contact sensitivity settings (87, 88). The Langerhans cell is efficient at capture and presentation of contact irritants, but this process can culminate in anergy and/or deletion of responding CD8^+^ T cells, with induction of ICOS^+^CD4^+^Foxp3^+^ regulatory T cell responses (89). The resident CD141^+^ dermal dendritic cell in humans can also effect tolerance through their expression of the inhibitory receptor ILT3 and of IL-10, which together upregulate CD25^+^ regulatory T cells that protect against allograft rejection (90). Migratory CD103^+^langerin^+^ dermal dendritic cells can also induce CD25^+^Foxp3^+^ Treg outgrowth from naïve T cells, at least in part through their expression of TGFβ (91).

### Hepatic dendritic and other tolerance-promoting cells

It is well recognized that operational tolerance occurs more frequently with liver transplants than with other organs, suggesting that this organ may have a unique tolerogenic capacity (92). Human liver dendritic cells comprise most prevalently BDCA-1^+^ DC that, unlike blood dendritic cells, secrete substantial amounts of IL-10 on TLR ligation, and this contributes to their high level induction of CD25^+^Foxp3^+^ Treg (11). It has also been reported that, in the steady state, hepatic myeloid and plasmacytoid dendritic cells can both induce tolerogenic T cell responses, although by distinct mechanisms – the myeloid cells express a mature phenotype and produce high levels of regulatory factors such as IL-10, IL-27, retinoic acid, and prostaglandin E2 (93–96), whereas hepatic plasmacytoid dendritic cells express a more immature phenotype and secrete high levels of IL-10 (97, 98). The non-parenchymal hepatic stellate cell, the major storage site for retinol in the body (99), would potentially also play a role in hepatic tolerance through provision of retinoic acid and thereby by-stander contributions to hepatic regulatory T cell induction (100, 101). Another factor to consider in hepatic tolerance is the resident liver macrophage, the Kuppfer cell. Kuppfer cells are present in very large numbers in the liver and express MHCII and co-stimulatory molecules, although as quiescent cells they only poorly present antigen. Nevertheless, just as the hepatic stellate cells are a rich source of retinoic acid, Kuppfer cells constitutively express abundant prostaglandin E2 and 15d-prostaglandin J2, which strongly inhibit T cell responses to immunostimulatory dendritic cells (102). Thus, there are multiple mechanisms that may contribute to the innate tolerogenic phenotype of the liver.

## Experimental Application of Tolerogenic Dendritic Cells

### Steady-state and immature dendritic cells

For practical reasons it is unlikely that steady-state dendritic cells freshly purified from donor tissues would be used clinically, but investigations into such cells have provided substantial insights into the immunobiology of tolerogenic dendritic cells. Steady-state dendritic cells from lymphoid organs (103, 104) and non-inflamed tissues (91, 104, 105) express a relatively immature phenotype – in general, such cells are tolerogenic (105, 106). For example, treatment with small numbers of antigen-pulsed steady-state CD8α^+^ splenic dendritic cells can induce asthma tolerance in mouse models, reversing the asthmatic animals’ bronchial hyperresponsiveness and airway eosinophil and Th2 cytokine recall responses to allergen challenge; expression of IL-10, TGFβ, and IDO, as well as direct dendritic cell–Teff cell contact each contribute to the tolerogenic activities of these cells (107). It is important to their activity that such steady-state dendritic cells remain quiescent while being purified or manipulated *ex vivo*, as even overnight exposure of CD8α^+^ dendritic cells to GM-CSF, for example, converts them into potent inducers of cytotoxic CD8^+^ T cell responses (108). Steady-state CD8α^+^ dendritic cell signaling leads to attenuated IL-2 expression by T cells and increased apoptosis, at least in part through the dendritic cell’s expression of FasL (109–111).

Tissue dendritic cells that acquire antigens *in situ* in such a way that they do not become activated also remain tolerogenic. Thus, as noted, steady-state airway mucosal dendritic cells routinely migrate to the draining lymph nodes and present innocuous allergens in a tolerogenic fashion – indeed, this is the default mechanism by which ≈80% of the human population remains allergen-tolerant (112). Similarly, targeting antigens to dendritic cells with anti-DEC205, for example, does not activate the cells and thus leads to antigen-specific tolerance in multiple models (113–115). And dendritic cells that phagocytose apoptotic cells remain in a largely quiescent state and thus are also tolerogenic (116, 117), at least in part via induction of TGFβ expression in the draining lymph nodes with consequent activation of Foxp3^+^ Treg (118).

There is also a large body of data regarding the tolerogenic properties of immunologically immature dendritic cells that have been generated *in vitro* from bone marrow or blood of mice or humans. These cells tend to express low levels of MHCII and co-stimulatory markers and have thus been thought of as largely ineffective in activating T cells through the classical TCR signaling pathways (119–122), although it has also been suggested that PD-L1 and PD-L2 expression by these cells contributes to their tolerogenic activities (123). There are ≈100 genes that are differentially expressed in immature versus immunostimulatory mouse bone marrow-derived dendritic cells, including a number of cytokines (e.g., Flt3L, TNF), chemokines (e.g., MIP2, RANTES), chemokine receptors (e.g., CCR2, CCR5), and other (e.g., RP105, Ax1) markers (124). Passive transfer of antigen-pulsed immature dendritic cells has been shown to induce tolerance either *in vivo* or *in vitro* in numerous experimental models and with human cells (125–132). An important caveat with use of immature dendritic cells to treat overtly inflammatory conditions is that the pro-inflammatory environment they face *in vivo* can activate these cells, such that they activate pathogenic (e.g., Th1, Th17) as opposed to regulatory T cell responses (133, 134), as discussed below.

## Induced Tolerogenic Dendritic Cells

Some of the first insights into the induction of a tolerogenic phenotype within dendritic cells arose from the studies of Langerhans cells that had been exposed to either ultraviolet B radiation (8) or IL-10 (8, 9). Dendritic cells from IL-10-expressing melanoma tumors (135) and IL-10-exposed immature monocyte-derived dendritic cells (136) were then also shown to be tolerogenic. This potential for using tolerogenic cells, whether dendritic cells or subsequently induced regulatory T cells, to dampen pathogenic responses has burgeoned into a field of immunology into itself. We now know that a large array of mediators can induce a tolerogenic phenotype within dendritic cell populations. These include IL-10 (9, 30, 33, 135–149) and other cytokines (150–158), corticosteroids (143, 159–162), vitamin D3 (160, 163–172), rapamycin (143, 160, 173–175), and neuropeptides (176, 177) (Table 1), each of which we will discuss. Although we will not discuss the following populations, it has been reported that dendritic cells can also be rendered tolerogenic by exposure to: anti-CD3 (178); *Aspergillus oryzae* protease (162); aspirin (179); atorvastatin (180); butyric or mycophenolic acids (181); the α7β0 isoform of C4b-binding protein (182); the FasL decoy receptor, decoy receptor-3 (183); galectin-1 (184, 185); growth-related oncogene (GRO)-gamma (186); intravenous immunoglobulin (IVIg) (187, 188); protein kinase C inhibitors (189); or retinoic acid (190–193), or by inhibition of miRNA let-7i (194). IL-10-, vitamin D3-, dexamethasone-, and rapamycin-induced tolerogenic dendritic cells stand out as populations that have had been particularly well-studied in mouse and/or human systems, so we will concentrate our discussions on these cells, with the interested reader referred to the cited reports for these alternate populations. Furthermore, given the potential ethical issues with use of dendritic cells transfected with viruses that express tolerogenic molecules (e.g., IL-10, CTLA4Ig) or that suppress stimulatory molecules (e.g., co-stimulatory, immunostimulatory, or pro-apoptotic molecules, such as CD80, IL-12, or TRAIL, respectively (195)), we will not devote significant discussion to these approaches at this time.

**Table 1:** **Phenotypes of human tolerogenic dendritic cells differentiated using different agents**.

Agent	DC	DCreg markers	Effector	Mechanisms of tolerance (outcomes)	Treg induced	Reference
Nil	Immature MDDC	↓ Co-stim, MHCII, IL-12	↓ Co-stim, MHCII	Induction of T cell anergy	N.D.	(119–122)
Apoptot. cells	Immature MDDC	↓ Co-stim, MHCII, IL-12	↓ Co-stim, MHCII	Induction of T cell anergy	Foxp3^+^ Treg	(116, 118)
		↑ TGFβ	
IL-10	Semi-mature MDDC (“DC10”)	↓ Co-stim, MHCII, IL-12	IL-10 and contact-depend	↓ Autol. T cell prolif.	CD25^+^Foxp3^+^ Treg	(30, 143, 196–198)
		↑ IL-10, ILT-2, -3, and -4, PD-L1 and -L2, GILZ	
	Immature MDDC	↓ Co-stim, MHCII	N.D.	↓ Allo. T cell prolif.	N.D.	(136, 158, 164)
		↑ ILT3, IL-10, GILZ, TLR2	
	Immature MDDC (“DC-10”)	↓ Co-stim, MHCII	IL-10, ILT4, HLA-G	↓ Allo. T cell prolif.	Tr1	(33)
		↑ ILT-2, -3, -4, HLA-G		↑ Tr1	
Vit D3	Immat. MDDC	↓ Co-stim and CD83, MHCII	N.D.	↓ Allo. T cell prolif.	Not CD25^+^ Foxp3^+^ Treg	(160)
		↑ HLA-DR	
	MDDC + LPS	Intermed co-stim/MHCII	PD-L1	↓ Allo. T cell prolif., Teff > IL-10 Treg	CD25^+^Foxp3^+^ Treg or ND	(163, 170–172, 199)
		↑ IL-10, TNF, PD-L1 and ILT3	
	MDDC + TLR stim.	hMDDC, LPS maturation	LPS ≫ IL-10 med	↓ Allo. T cell prolif.	N.D.	(164)
	MDDC + LPS	↑ Surface TNF	Surface TNF	↑ Treg induction	N.D.	(167)
		↓ Secr. TNF	
	Dermal DC	N.D.	IL-10	↓ Allo. T cell prolif.	Tr1 cells	(200)
	Langerhans cells	N.D.	TGFβ	↓ Allo. T cell prolif.	CD25^+^Foxp3^+^ Treg	(200)
	CD141^−^CD1c^+^ blood DC	↑ CD83	IL-10		CD25^+^Foxp3^+^ Treg	(90)
		↑ CD141, CD14, ILT3, MØ mann. R	
Dex	Immat. MDDC	↑ CD86, MHCII	IL-10	↓ Allo. T cell prolif.	N.D.	(160)
		CD83 med	
	MDDC + LPS	Intermed co-stim/MHCII ↑ IL-10	N.D.	↓ Allo. T cell prolif., Teff > IL-10 Treg	IL-10-secreting, contact-depend. Treg	(171)
	MDDC ± TLR stim.	Intermed co-stim/MHCII	N.D.	↓ Allo. T cell prolif.		(164)
		ILT3^+^, IL-10^+^, GILZ^+^, TLR2^+^	
	DC2.4 cells	↓ IL-12	N.D.	↓ Allo. T cell prolif.	CD25^+^Foxp3^+^ Treg	(201)
Steroid	MDDC	GILZ^+^	N.D.		N.D.	(202)
VitD3 + Dex	MDDC	↓ Co-stim and CD83, MHCII	IL-10	↓ Allo. T cell prolif.	Tr1 or N.D.	(165, 203)
		> CD14, HLA-DR, CD80, CD273		↓ CD25^+^Foxp3^+^ Treg	
				↑ Tr1 and Breg	
VIP-DC	Immature MDDC	↓ Co-stim, MHCII ↑ IL-10	N.D.	Weak naïve allo T cell activation	Tr1 and CD4^+^ CD28^−^CTLA4^+^ Treg	(176, 204)
Rapamycin	Immat. MDDC	↓ Co-stim med. MHCII	IL-10?	↓ Allo. T cell prolif.	CD25^+^Fopx3^+^ Treg	(160, 205, 206)
				↑ Foxp3^+^CD25^+^ Treg	

### Interleukin-10-induced regulatory dendritic cells

As noted, IL-10 was one of the first mediators shown to induce human dendritic cells to adopt a tolerogenic phenotype (8, 9, 122, 135, 136, 141, 148, 150, 209–212). These reports together indicated that IL-10-differentiated monocyte-derived dendritic cells display reduced levels of MHCII and co-stimulatory markers, and can induce Teff cell anergy. Sometime later it was shown that IL-10-induced semi-mature CD14^+^ monocyte-derived dendritic cells (DC10) from atopic asthmatic individuals suppress specific allergen-driven proliferative and Th2 cytokine responses of autologous peripheral blood CD4^+^CD25^−/lo^Foxp3^−^Teff cells, and convert these Teff cells into regulatory T cells (30). The maturation status of these DC10 was attributable to their exposure during differentiation to a stimulatory cocktail containing IL-1β, TNF, IL-6, and PGE2, in addition to IL-10 (30), but these cells are resistant to further, LPS-induced, maturation (209). DC10 express low levels of MHCII, co-stimulatory markers, 4-1BBL and OX40L, but they strongly express DEC205, IFNα1, CCR7, ILT2 (an inhibitory HLA-G receptor), as well as IL-10 (Table 2). They induce Teff cells to differentiate into IL-10-secreting CD25^+^Foxp3^+^LAG-3^+^CTLA4^+^ regulatory T cells, which in turn suppress allergen-driven responses of autologous Teff cells in a contact-dependent fashion (30). Others found that similar semi-mature IL-10-differentiated dendritic cells express high levels of ILT3, ILT4, PD-L1, and PD-L2, that they (but not immature cells) respond strongly to the lymph node-homing chemokine CCL19 (143), and that they induce regulatory T cells that also suppress allogeneic T cell responses in a contact, but not IL-10- or TGFβ-dependent fashion (196). These DC10 also express glucocorticoid-induced leucine zipper (GILZ), which is both necessary and sufficient for expression of IL-10, ILT3, and PD-L1 by these cells – GILZ silencing eliminates their tolerogenic activities (197, 198). IL-10-differentiated human monocyte-derived dendritic cells that have never been exposed to maturation-inducing agents are also tolerogenic (33, 148, 212). As noted above, a minor population of IL-10-producing circulating dendritic cells, called DC-10, was recently identified in humans (33), and those investigators also generated an analogous population of immature IL-10-differentiated dendritic cells (DC-10) that similarly express IL-10 (Table 2), as well as the inhibitory receptors ILT2, ILT3, ILT4, and HLA-G (33). Others have noted that such cells also express signaling lymphocyte activation molecule (SLAMF1, CD150) (148), which inhibits CD40-mediated signal transduction (213), and would therefore interfere with two-way dendritic cell-T cell conversations. These cells have been reported to suppress Teff cell responses in a manner that is contact-dependent, and independent of any role for secreted soluble mediators (148), although others note that IL-10 secretion and cell-surface inhibitory receptors are both important to the regulatory activities of such immature IL-10-differentiated dendritic cells (33). It is very intriguing that exposure of semi-mature human dendritic cells to IL-10 leads to their induction of classical CD25^+^Foxp3^+^ Treg (30, 196), while exposure of immature human dendritic cells to IL-10 leads to induction of Foxp3^−^ Tr1 cells (33). It will be interesting to determine whether exposure of such immature regulatory dendritic cells to inflammatory (i.e., maturation-promoting) conditions would qualitatively or quantitatively affect their immunobiology.

**Table 2:** **Impact of phenotype on the levels of IL-10 secretion by regulatory dendritic cells**.

Differentiating agent	DC (IL-10 levels)	Reference
**NON-REGULATORY DENDRITIC CELLS**		
TNF	Semi-mature MDDC (≈35 pg/ml)	(30)
Nil	semi-mature MDDC (LPS, >700 pg/ml; CD40L, >2 ng/ml)	(207)
**TOLEROGENIC DENDRITIC CELLS**		
Vitamin D3/dexamethasone	MDDC (9 ng/ml)	(203)
C1Q	MDDC (5 ng/ml)	(208)
Vasoactive intestinal peptide	MDDC (LPS, ≈5 ng/ml)	(176)
Galectin-1	MDDC (LPS, ≈500 pg/ml)	(185)
Vitamin D3	Dermal DC (CD40L, ≈300–700 pg/ml)	(90)
	MDDC (unstim or LPS, ≈100 pg/ml)	(160, 164)
	MDDC (LPS or CD40L, ≈2 ng/ml)	(167)
	MDDC (CD40L, 4 ng/ml)	(163)
IL-10	Immat. MDDC (unstim, 200–750 pg/ml; CD40L, 1.5 ng/ml)	(33, 143, 163, 164)
	Semi-mature MDDC (unstim, 300 pg/ml; LPS, 7 ng/ml)	(30, 158)
Dexamethasone	Immat MDDC (unstim, 25–200 pg/ml)	(143, 159, 160, 198)
	MDDC (LPS or CD40L, 0.5–3 ng/ml)	(159 –161, 198)
Rapamycin	MDDC (unstim or LPS, 50–100 pg/ml)	(143, 160)
TGFβ	MDDC (unstim, 200 pg/ml; LPS, ≈2 ng/ml)	(143, 158)

Murine DC10 can prevent the onset of asthma in experimental mice, as well as reverse the asthmatic phenotype in severely affected animals (137, 138, 140, 214–216), just as do dendritic cells that have been virally transfected to express very high levels of IL-10 (146). These DC10, which are not exposed to maturational stimuli during differentiation, display low levels of cell-surface MCHII and co-stimulatory markers, are avidly phagocytic and chemotactically responsive to MIP-1α, and express elevated levels of IL-10, TGFβ (137, 138, 215), and PD-L1 (Li et al., unpublished observation). They are highly effective therapeutically in mouse models of ovalbumin (OVA) – (138, 140, 214–216) and house dust mite – (137) asthma. In both settings, DC10 abrogate airway hyperresponsiveness (AHR) within 3 weeks of treatment and dampen the allergic Th2 phenotype in an allergen-specific fashion (137, 138, 140, 215). This suppression of allergen-induced airway eosinophil and Th2 cytokine responses and circulating allergen-specific IgE and IgG1 levels is progressive, such that at 8 months after a single DC10 treatment these parameters are at near background levels (138), although four DC10 treatments bring the asthma phenotype to near background within 2 months (138). Cell tracking studies indicate that DC10 that are delivered intraperitoneally accumulate maximally in the lungs and lung-draining lymph nodes within 1 week, but few, if any, DC10 can be detected within any anatomic compartment at 3 weeks post-treatment (214). This indicates that while tolerance induced by DC10 is long-lived, most of its impact is realized only after the treatment cells have disappeared from the body. That is consistent with the observation that DC10 treatments induce CD4^+^CD44^hi^CD69^hi^CD62L^lo^CD25^lo^Foxp3^−^ Teff cells to transdifferentiate into CD4^+^CD25^+^Foxp3^+^ Treg, with maximal Treg activation occurring at 3 weeks after DC10 treatment (215). Human DC10-induced CD25^+^Foxp3^+^ Treg express LAG3 and CTLA3 (30), while the analogous Treg in DC10-treated asthmatic mice express LAG3, cytotoxic T lymphocyte antigen-4 (CTLA4) (137, 215), ICOS, PD-1, GITR (215), and neuropilin-1, but lower levels of Helios (217). Infectious tolerance is also evident in these animals, as the endogenous pulmonary CD11c^+^ dendritic cells of DC10-treated asthmatic animals also take on a regulatory phenotype (Li et al., unpublished observation). While DC10 engage CD4^+^CD25^+^Foxp3^+^ natural (n)Treg in a productive fashion and these T cells have a modest role in the asthma tolerance within DC10-treated animals, DC10-induced CD25^+^Foxp3^+^ (i)Treg are many-fold more effective than naturally occurring CD25^+^Foxp3^+^ regulatory T cells (nTreg) of identical TCR specificity in suppressing the asthma phenotype (217).

IL-10 expression by immature or otherwise quiescent dendritic cells has been reported numerous times to be important to tolerance induced by these cells (24, 107, 218), and DC10 (as well as DC-10) express yet higher levels of this regulatory cytokine (30, 33, 137, 138, 140, 215, 216) (Table 2). Indeed, expression of IL-10 by DC10 is critical (140, 214) although not sufficient for tolerance induction, inasmuch as MHCII-knock-out DC10, which expresses otherwise therapeutic levels of IL-10, do not induce tolerance (214). Moreover, combined IL-10 and MHCII expression by DC10 is still not sufficient for full expression of tolerance – allergen-presenting CD80/CD86 double knock-out (214) or CD40-knock-out (W. Dawicki, H. Huang and J.R. Gordon, unpublished observations) DC10 still do not induce tolerance at levels equivalent to wild-type DC10 (214). This underscores that conversion of Teff cells to regulatory T cells by DC10 requires not only delivery of tolerogenic signals to the T cell, but also productive feedback from the engaged T cell to the DC10.

### Vitamin D3-induced regulatory dendritic cells

Vitamin D and its metabolites would appear to have a significant influence within the immune system, such that there is substantial evidence of an unrealized potential for its use in an array of immunologic disorders [reviewed in Ref. (219, 220)]. It is clear that vitamin D3 can induce differentiation of tolerogenic dendritic cells (DC-VitD3) (163, 170, 221, 222). Addition of vitamin D3 to mouse bone marrow (177, 223, 224) or human monocyte-derived (164, 170, 172, 225) dendritic cell cultures induces cells that express low levels of MHC II and co-stimulatory molecules, and produce IL-10 instead of IL-12 (Table 2). Semi-mature monocyte-derived DC-VitD3 express augmented levels of TNF and PDL-1, and this PDL-1 is reportedly critical to their induction of IL-10-expressing contact-dependent Treg (171), as is expression of membrane-bound TNF by these dendritic cells (226). As with IL-10-differentiated dendritic cells, DC-VitD3 only respond to the lymph node-homing chemokine CCL19 if they have been exposed to maturational stimuli (143). This again raises the question of whether such chemokine-dependent lymph node homing might reasonably be expected to contribute, if not be critical, to the tolerogenic activities of regulatory dendritic cells. Addition of vitamin D3 to cultures of human skin Langerhans cells leads to expression of TGFβ by these cells and thereby downstream induction of CD25^hi^CD127^lo^Foxp3^+^ cells (i.e., classical inducible Treg) (200). It similarly induces CD141^−^CD1c^+^ human blood dendritic cells to differentiate into IL-10-expressing dermal dendritic cell-like CD141^+^CD14^+^ILT3^+^ cells that induce development of CD25^hi^CTLA4^+^Foxp3^+^ Treg responses (90). In contrast, addition of vitamin D3 to cultures of human dermal dendritic cells upregulates expression of IL-10 and their induction of IL-10-expressing Foxp3^−^ Tr1 cells (200). This highlights again that exposure of different dendritic cell populations to the same mediator can have very divergent outcomes in terms of the type(s) of regulatory T cells so induced. DC-VitD3 have been shown to be tolerogenic *in vivo* as well. Treatment of diabetic mice with pancreatic islet antigen-pulsed DC-VitD3 prior to pancreatic islet transplantation significantly decreases subsequent islet rejection (166), while sensitization of mice with H-Y antigen-pulsed DC-VitD3 leads to prolongation of male skin grafts in female recipients (177, 223, 224).

### Dexamethasone-induced tolerogenic dendritic cells

The anti-inflammatory and immunosuppressive properties of corticosteroids have been known and employed clinically since their discovery some 75 years ago (227). While glucocorticoid treatments have significant clinical benefits in terms of suppressing inflammation, and it has been shown that they increase the numbers of CD4^+^CD25^hi^ cells and Foxp3 expression levels in multiple inflammatory settings, these increases are not necessarily associated with augmented Treg activity (228). Corticosteroids do induce immature dendritic cells to adopt a tolerogenic phenotype and thereby contribute to the anti-inflammatory properties of these agents (159, 229, 230), but the fact that mature dendritic cells undergo apoptosis in response to *in vitro* or *in vivo* dexamethasone treatment suggests that its effects on dendritic cells are somewhat more complex (231). Dendritic cells that are differentiated in the presence of dexamethasone (DC-Dex) express low levels of co-stimulatory markers and MHC II, produce elevated levels of IL-10 and less IL-12 (159, 161, 164, 171, 229, 230, 232), and express modestly elevated levels of ILT2 (198) and ILT3, but high levels of GILZ (164). As with semi-mature IL-10-differentiated dendritic cells, GILZ expression by DC-Dex is critical to their expression of IL-10, ILT3, and B7-H1/PDL-1 (197); both populations also maintain their immunosuppressive phenotype even after stimulation with TLR4 agonists (209, 233, 234). The similarities between DC10 and dexamethasone-conditioned dendritic cells extends further – dexamethasone-exposed DC2.4 dendritic cells also induce Foxp3^+^ Treg differentiation *in vitro* (201), while use of DC-Dex immunotherapy for experimental corneal allografts similarly leads to increased tissue levels of intragraft Foxp3^+^ T cells, reduced levels of graft inflammatory cell infiltrates, and prolonged graft survival (235). And others have reported that repetitive stimulation of T cells with DC-Dex induces the T cells to adopt a contact-dependent regulatory T cell phenotype (171). DC-Dex treatment of murine recipients of MHC-mismatched heart transplants leads to delayed rejection of the allografts (234) although, oddly, DC-Dex treatments reportedly accelerate antibody-mediated graft rejection responses to transplanted MHC-mismatched pancreatic islets in rats (236). Interestingly, the contact-dependent regulatory T cells induced by DC-Dex, but not those induced by DC-VitD3, reportedly suppress T cell responses in an antigen-independent fashion (171), although others have shown that, as a general feature, activated regulatory T cells readily suppress by-stander Teff cell responses (237, 238).

### Vitamin D3 and dexamethasone-induced tolerogenic dendritic cells

While vitamin D3 and dexamethasone each can induce a tolerogenic phenotype in dendritic cells, some investigators have further assessed the regulatory activities of cells generated in the presence of both vitamin D3 and dexamethasone (DC-Dex/VitD3). DC-Dex/VitD3 produce much high levels of IL-10 (i.e., 9 ng/ml) (203) than either DC-VitD3 or DC-Dex (i.e., 0.1–4 ng/ml) (Table 2) (143, 159, 160, 163, 164, 167, 198), and thus display a higher IL-10/IL-12 expression ratio and poorly stimulate allogeneic T cell proliferation responses (165). They reportedly cannot effectively prime naïve CD8 T cells but, interestingly, while a single DC-Dex/VitD3 treatment drives expansion of memory CD8 T cells, any subsequent DC-Dex/VitD3 exposure leads to collapse of the CD8^+^ T cell populations (239). DC-Dex/VitD3 have been shown to be somewhat effective in suppressing colitis pathology in a mouse model, apparently also in an antigen-independent manner (240).

### Neuropeptide-induced tolerogenic dendritic cells

Vasoactive intestinal peptide (VIP) is a 28-amino acid immunomodulatory neuropeptide that binds to B-class G-protein-coupled receptors such as the VPAC1 and VPAC2 (241, 242). VIP treatments induce regulatory T cell responses in experimental animals and with human Teff cells (243). For example, VIP treatment of mice with TNBS-induced colitis induces tolerance responses, dampening TLR2- and TLR4-induced inflammation and increasing expression of Foxp3 and TGFβ (244), as it does in a rat model of collagen-induced arthritis (245). But VIP can act directly on the Teff cells – culture of CD25^−^Foxp3^−^ Teff with VIP induces their differentiation into CD25^+^Foxp3^+^ Treg that express high levels of IL-10 and CTLA4 and are protective in a mouse model of graft versus host disease (GVHD) (246). Nevertheless, VIP can also induce dendritic cells to adopt a regulatory phenotype and thereby affect tolerance by this means. Differentiation of human dendritic cells in the presence of VIP (DC-VIP) or the neuropeptide pituitary adenylate cyclase-activating polypeptide (PACAP) induces the development of cells that secrete of high levels of IL-10, and strongly induce regulatory T cell responses. DC-VIP treatments dampen pathology in a number of experimental settings, including experimental allergic encephalomyelitis (EAE), rheumatoid arthritis (247), bone marrow transplant-induced GVHD (248), and colitis (249). While a number of reports indicate that DC-VIP induce Tr1 phenotype regulatory cells, as determined by secretion of IL-10/TGFβ, but not IFNγ, IL-2, IL-4, or IL-5 (204, 250–252), other reports indicate that DC-VIP instead induce CD4^+^CD25^+^Foxp3^+^ Treg responses (253–255) in some of the same model systems. DC-VIP can also induce IL-10-secreting CD28^−^CTLA4^+^CD8^+^ Treg (176, 252). VIP-secreting VIP-lentivirus-transfected DC are similarly tolerogenic in mouse models of acute and chronic EAE and cecal ligation-and-puncture sepsis (177). It has been speculated that DC-VIP would be more effective therapeutically when targeting Th1 rather than Th2 responses (176), ostensibly because VIP skews Th1 or Th17 T cells to a Th2 phenotype (256). This raises an important question in dendritic cell immunotherapeutics, and that is whether the specific type of regulatory cell to be employed (e.g., DC-Dex versus DC-VIP) needs to be carefully matched with, for example, the Th1, Th2, or Th17 nature of the target disease in order to ensure optimized outcomes.

### Rapamycin-induced tolerogenic dendritic cells

Rapamycin is a macrolide immunosuppressive agent that dampens dendritic cell maturation through binding to the serine/threonine protein kinase mammalian target of rapamycin (mTOR). Signaling via mTOR has broad-ranging effects in many systems, including the nervous system, nutrition, and others, where it regulates cell growth, proliferation, motility, and survival (257). Antigen recognition by naïve CD4^+^ and CD8^+^ T cells activates mTOR and thereby fosters cellular progression to a committed Foxp3^−^ Teff phenotype (205), while suppression of mTOR with rapamycin leads instead to induction of fully functional CD25^+^Foxp3^+^ Treg (258). Thus it was reported some time ago that rapamycin increases the regulatory activities of CD4^+^CD25^+^Foxp3^+^ Treg (206). Clinically, rapamycin has been widely used to prevent allograft rejection, particularly in renal transplant patients (206), although the potential for rapamycin-related adverse cutaneous manifestations in these patients has limited its broad applicability (259). Rapamycin affects both T cells and dendritic cells, although it displays divergent effects on myeloid and monocyte-derived dendritic cells, augmenting the allostimulatory capacity of the former cells but markedly dampening the immunostimulatory phenotype of monocyte-derived dendritic cells (260). In experimental systems rapamycin treatments impair Flt3L mobilization of murine dendritic cells, their upregulation of co-stimulatory molecule and inflammatory cytokine expression, and their allostimulatory activity (261), even after exposure to activating agents such as LPS or anti-CD40 (262). Mouse dendritic cells that are differentiated in the presence of rapamycin (DC-Rap) induce naïve T cells to differentiate into CD25^+^Foxp3^+^ Treg (263). Moreover, such DC-Rap enhance apoptotic death among alloreactive CD8 T cells (264), further contributing to the tolerance response of transplant recipients. DC-Rap treatment of murine heart transplant recipients similarly induces outgrowth within the transplants of Foxp3^+^ Treg and, as a consequence, long term organ survival (262), just as has been shown in numerous other studies (173, 206, 261, 262, 265, 266).

## Impact of Delivery Route and Inflammation on Therapeutic Outcomes

### Selecting the correct delivery route for tolerogenic dendritic cells

Not all routes for delivery of tolerogenic dendritic cells will necessarily provide the desired outcomes. For example, we reported that CD45.2^+^ DC10 that are delivered intraperitoneally to congenic CD45.1^+^ mice with a severe asthma phenotype appear within the lungs and airways of recipient mice within 2 days of delivery, achieve maximal numbers in this compartment by 7 days and then wane thereafter. DC10 appear in the lung-draining (mediastinal) lymph node of these animals in lower numbers, but with approximately the same kinetics, and also in the spleen but not cervical nodes, MLNs, blood, bone marrow, or liver. Within 3 weeks of delivery the treatment cells are no longer detectable in the lungs or mediastinal lymph nodes (214), suggesting that the natural lifespan of such DC10 may be 2–3 weeks. We know that DC10 treatments correct ~50% of the pathognomic bronchial hyperresponsiveness seen in asthma phenotype mice within 2 weeks of treatment and that by 3 weeks this airway response is completely normalized (138). Moreover, the time of maximal activation of regulatory T cells in the lungs of these animals is 3 weeks after DC10 delivery, but it was not determined whether the primary site within which the DC10 induce Teff cells to differentiate into regulatory T cells was *in situ* in the lungs or in the mediastinal lymph nodes (or both) (215). This remains an important, but unanswered question.

We also assessed the relative effects of intraperitoneal (i.p.), transtracheal (t.t.), subcutaneous (back skin; s.c.), or intravenous (i.v.) DC10 delivery to asthmatic animals and found that i.p. or t.t. delivery were equally effective, fully reversing bronchial hyperresponsiveness, and rapidly dampening airway eosinophil and Th2 cytokine responses to allergen challenge and circulating allergen-specific IgE and IgG1 levels (138). The s.c. DC10 treatments dampened the airway recall responses to allergen challenge, but not bronchial hyperresponsiveness, nor did they significantly reduce systemic IgE levels (138). On the other hand, multiple investigators have reported that s.c. delivery of tolerogenic dendritic cells is protective in rat models of EAE (267–271), which suggests that the anatomic site of the target pathology in immunotherapeutic applications may be important in selecting the delivery route for the treatment dendritic cells. Intravenous delivery of DC10 has no discernible impact of the disease phenotype in a mouse model of asthma (138, 272) or a rat model of EAE (271), but in mouse models of cardiomyopathy (147), experimental immune myocarditis (149, 273), and diabetes (274, 275) i.v. delivery of tolerogenic dendritic cells significantly reduces local pathology and induces tolerance. Similarly, i.v. infusion of DC-VitD/IL-10 in a rhesus macaque model of allogeneic kidney transplantation significantly prolonged survival relative to control animals (rapamycin/CTLA4Ig treatment, but no dendritic cells) (276). There has not been a sufficient number of comprehensive studies on the impact of the route of dendritic cell delivery on tolerance outcomes to generate specific guidelines at this point in time, but it does appear that the disease or compartment being targeted may be an important consideration. Certainly, we would expect that the cells should be migration-competent (i.e., express appropriate chemokine receptors), such that they are able to travel to the disease target site or its draining lymph nodes in order to best interact with the cognate Teff cells.

### Use of tolerogenic dendritic cells in inflammatory settings

An important consideration in clinical use of tolerogenic dendritic cells, particularly when targeting inflammatory diseases (e.g., colitis, inflammatory bowel disease), is whether pre-existing adverse conditions that these cells might encounter after delivery can alter or ablate their tolerogenic activity. If so, could an inflammatory milieu convert the treatment dendritic cells into immunostimulatory populations that might exacerbate rather than ameliorate disease severity? While immature dendritic cells can have substantial tolerogenic activities, we know that exposure of these cells (133, 134) or even some populations of semi-mature dendritic cells (133, 134) to inflammatory environments can induce them to differentiate into potently immunostimulatory cells that *augment* disease severity. With this in mind, many investigators have assessed the impact of maturation-provoking (30, 90, 143, 165, 197) or otherwise inflammatory (163, 164, 167, 170, 171, 177, 222) signals on the tolerogenic phenotype of their differentiated dendritic cells. Dendritic cells express receptors for and can be activated by a number of pro-inflammatory cytokines (e.g., IL-1, TNF, IFN, TSLP) (277) and they can express numerous pattern recognition receptors [e.g., protease-activated receptors (PARs), TLR, C-type lectin receptors (78, 164, 278–281)], retinoic acid-inducible gene-1 (RIG-1) and the melanoma differentiation-associated gene-5 (MDA-5) (281), through which they interact with microbial and non-microbial agents. For example, a number of “natural” allergens (e.g., house dust mite) trigger inflammatory responses through their abilities to activate cells via PAR2 (282, 283) or C-type lectin receptors such as DC-SIGN and dectin-2 (284), while TLR signaling can potently activate expression of inflammatory signals by immature or mature dendritic cells. There have been a number of excellent reviews that address the expression of TLR by human and mouse dendritic cells [e.g., Ref. (281)], such that we will not address this issue herein.

Toll-like receptor signaling within tolerogenic dendritic cell populations does not always have a detrimental outcome. For example, BDCA-1^+^ human liver dendritic cells secrete substantial amounts of IL-10 on TLR ligation, and this contributes to their high level induction of CD25^+^Foxp3^+^ Treg (11). Human DC10, DC-Dex, and DC-VitD express the same panel of TLR as monocyte-derived dendritic cells, such that all are responsive to Pam3CSK4, polyinosinic-polycytidylic acid, LPS, and flagellin (164), but the tolerogenic populations uniquely upregulate expression of TLR2 on TLR engagement (164). Moreover, TLR2 or TLR4 signaling in human DC-VitD3 and DC-Dex induces expression of the tolerance-promoting cytokines IL-10 and IL-27 (160, 285). Others have reported that human DC-Dex are refractory to challenge with an array of heat-killed gram-negative bacteria (e.g., *Escherichia coli, Protheus mirabillis, Klebsiella pneumoniae, Salmonella thyphimurium*) (286), while DC-Rap (160) and DC-VIP (252) are resistant to reversal of their tolerogenic phenotype by LPS challenge. Interestingly, while isolated LPS challenge induces an IL-12 response by immature monocyte-derived dendritic cells, simultaneous exposure of these cells to LPS and IFNγ reportedly leads to a transient IL-12 response that is replaced within 24–48 h with a robust IL-10 response (287).

Finally, while we may well be able to design and generate tolerogenic dendritic cells that are resistant to reversal of phenotype by inflammatory environments, it is clear that the tolerance they induce is also dependent on transference of that phenotype to the regulatory T cells with which they interact. Moreover, infectious tolerance also involves the conversion of endogenous tissue dendritic cells into tolerogenic populations by the induced regulatory T cells (226, 288). Indeed, it has been suggested that a defect in such infectious tolerance processes may contribute to the development of an asthma phenotype in affected individuals (289). The desired outcome in dendritic cell immunotherapy is the induction of regulatory T cells that can reverse pathogenic Teff cell responses, but at least some populations of regulatory T cells can be converted into pathogenic Teff cells in the context of inflammatory environments – it has been shown that Foxp3^+^ Treg can convert to Th17 cells in animals with colitis (290, 291), but we seem to have only scant evidence regarding the extent to which other populations of regulatory cells (e.g., Tr1 or Th3 cells) can be enticed to such reversal of phenoytpe *in vivo*. In considering whether inflammatory environments may differentially affect the phenotype of regulatory T cells (or dendritic cells), we query whether the regulatory T cells that are naturally associated with a specific compartment (e.g., Th3 cells in the gut) might be more resistant to reversal of phenotype by challenges they would routinely encounter in that compartment than other regulatory T cells (e.g., Treg, Tr1). Finally, we raise the issue of whether in some specific settings, it might be advisable to activate multiple types of regulatory T cell responses, such that the tolerance so induced might be less susceptible by reversal by subsequent coincidental inflammatory events.

## Clinical Application of Tolerogenic Dendritic Cells

The first tolerogenic dendritic cell study in humans was undertaken by Ralph Steinman’s lab. They demonstrated that s.c. administration of antigen-loaded immature dendritic cells (2 × 10^6^ cells/subject) was well tolerated by the study subjects and also that the treatments could suppress antigen-specific CD8^+^ T cell responses (128) for ≤6 months (127). More recently a clinical trial was undertaken with 10 subjects with type 1 diabetes, each of whom was given 1 × 10^7^ autologous dendritic cells intradermally four times at 2 week intervals; the treatment cells had been transduced with anti-sense oligonucleotides to silence co-stimulatory molecules (i.e., CD40, CD80, and CD86), although efficacy data on that silencing was not reported (292). The authors had developed their silencing protocols in a mouse model of type 1 diabetes and shown that the dendritic cell treatments had had statistically significant, though quite modest, disease-sparing effects (293). As with the earlier study by Steinman (127, 128), there were no adverse events related to the dendritic cell treatments in this latter study, but there were few if any immunologically discernible tolerance outcomes attributable to the dendritic cell treatments (292).

There have been a large number of *in vitro* studies performed as proof of principle that tolerogenic dendritic cells can efficiently reduce Teff cell responses in humans. As noted above, it was shown that semi-mature IL-10-differentiated dendritic cells (i.e., DC10) generated from atopic asthmatic donors can suppress the responses of autologous T cell to specific allergen. Moreover, the DC10 induce the outgrowth of immunosuppressive CD4^+^CD25^+^Foxp3^+^LAG3^+^CTLA4^+^ Treg from the peripheral blood Teff cell pool (30). Others have reported that DC-VitD/Dex from individuals with rheumatoid arthritis (294) or DC-VitD3 from subjects with relapsing-remitting multiple sclerosis (295) are both able to suppress autologous CD4^+^ Teff cell responses to specific antigen-presenting mature dendritic cells.

In conclusion, it is clear that multiple mediators can induce a tolerogenic phenotype in dendritic cells, and that these substantially influence the conversations that occur between the dendritic cell and naive or Teff cells. These tolerogenic dendritic cells employ both secreted mediators (e.g., IL-10, retinoic acid) and inhibitory receptors to drive regulatory T cell induction, but can also provide additional signals (e.g., integrins) to direct these nascent Treg to the appropriate anatomic compartment (Figure 1). A major challenge we will face in the application of such tolerogenic dendritic cells for immunotherapy will be to carefully match or optimize the type(s) of tolerogenic dendritic cells to be employed with the clinical targets and desired endpoints.

## Author Contributions

Wojciech Dawicki reviewed the literature for and wrote parts of the section on naturally occurring populations of dendritic cells, Yanna Ma contributed to the section on T cell biology, Laura Churchman wrote the introduction, Sara A. Gordon collated the literature on the different types of dendritic cells that have been reported, and John R. Gordon wrote the section on the different types of experimental dendritic cells that have been reported. All authors contributed to the planning and editorial phases of the review.

## Conflict of Interest Statement

The authors declare that the research was conducted in the absence of any commercial or financial relationships that could be construed as a potential conflict of interest.

## Abbreviations

AHR, airway hyperresponsiveness; APCs, antigen-presenting cells; BAL, bronchoalveolar lavage; BDCA, blood dendritic cell antigen; CTLA4, cytotoxic T lymphocyte antigen-4; DC10 or DC-10, semi-mature or immature, respectively, IL-10-differentiated dendritic cells; DC-SIGN, dendritic cell-specific intracellular adhesion molecule 3-grabbing non-integrin; Foxp3, the transcription factor forkhead box P3; ICOS and ICOS-L, inducible costimulator and inducible costimulator ligand, respectively; IDO, indoleamine-2,3-dioxygenase; ILT, immunoglobulin-like transcript; iTreg, induced CD25^+^Foxp3^+^ regulatory T cells; MHC, major histocompatibility complex; nTreg, naturally-occurring CD25^+^Foxp3^+^ regulatory T cells; OVA, ovalbumin; RALDH2, retinaldehyde dehydrogenase 2; Teff cell, effector T cell; TLR, toll-like receptor; Treg cell, regulatory T cell.
